# SY-SLAM: Real-Time Dynamic Indoor RGB-D SLAM with SuperPoint Detection and Asynchronous YOLOv8s-Based Keypoint Suppression

**DOI:** 10.3390/s26113315

**Published:** 2026-05-23

**Authors:** Shaoshuai Zhi, Shuangfeng Wei, Shan Zhou, Yulan Lao, Mingyang Zhai, Tianyu Yang, Keming Qu, Boyan Jiang

**Affiliations:** 1 School of Geomatics and Urban Spatial Informatics, Beijing University of Civil Engineering and Architecture, Beijing 102616, China; 202304050117@stu.bucea.edu.cn (S.Z.);; 2 Research Center of Representative Building and Architectural Heritage Database, Ministry of Education, Beijing 102616, China; 3 Beijing Key Laboratory for Architectural Heritage Fine Reconstruction & Health Monitoring, Beijing 102616, China; 4 School of Intelligent Science and Technology, Beijing University of Civil Engineering and Architecture, Beijing 102616, China

**Keywords:** visual SLAM, RGB-D camera, dynamic environments, learned keypoint detector, bounding-box-based keypoint suppression

## Abstract

Traditional visual SLAM pipelines are typically designed under the static-world assumption and often degrade severely in indoor environments with frequent human motion. To improve trajectory accuracy and front-end stability in such scenarios while maintaining real-time throughput, we present SY-SLAM, an RGB-D SLAM system for dynamic indoor environments with frequent human motion. (S stands for SuperPoint, which is used as a detector-only learned keypoint front-end, and Y stands for YOLO, which provides asynchronous person-aware keypoint suppression based on detected human bounding boxes.) We integrate a TensorRT-deployed detector-only SuperPoint module to improve keypoint repeatability and robustness while retaining ORB binary descriptors for efficient matching and place recognition within the ORB-SLAM3 framework. To avoid feature starvation while preserving keypoint quality, we further introduce an adaptive SuperPoint keypoint selection strategy that applies stricter filtering when keypoints are abundant and relaxes the selection constraints when they are scarce. In parallel, an asynchronous YOLOv8s TensorRT thread performs person detection with temporal bounding-box memory, and keypoints inside detected person regions are removed before ORB descriptor computation and matching to reduce dynamic-feature contamination in the front end. We evaluate SY-SLAM on five dynamic TUM RGB-D fr3 sequences using ATE and RPE metrics. Compared with ORB-SLAM3, SY-SLAM reduces ATE RMSE by 93.45% across four dynamic walking sequences. On the widely reported fr3/w/x sequence, SY-SLAM achieves competitive accuracy with recent dynamic SLAM methods while maintaining real-time performance. The system runs in real time at 46.8 Hz (21.36 ms per frame) on an Intel i9-13900H CPU with an NVIDIA RTX 4070 Laptop GPU.

## 1. Introduction

Simultaneous localization and mapping (SLAM) is a fundamental capability for autonomous robots and intelligent mobile systems, enabling them to localize themselves while incrementally building a map of an unknown environment. Among the various sensor modalities, visual and RGB-D SLAM have become particularly attractive because cameras provide rich scene information at relatively low cost and are widely applicable in indoor robotics, augmented reality, and service automation. Representative feature-based systems such as ORB-SLAM2 and ORB-SLAM3 have demonstrated strong accuracy, mature optimization backends, and practical real-time performance in static or mildly dynamic scenes [1,2]. However, these pipelines are still largely developed under the implicit static-world assumption. In indoor environments with frequent human motion, features extracted from moving people may enter the correspondence and pose-estimation process, leading to mismatches, trajectory drift, unstable tracking, and map contamination [3,4]. This limitation of the static-world assumption and the resulting robustness challenges in dynamic environments have also been discussed in recent survey papers on dynamic SLAM and robust visual SLAM [5,6].

To address this problem, many dynamic-scene SLAM methods incorporate semantic perception into conventional feature-based pipelines. Representative systems such as DynaSLAM and DS-SLAM use semantic segmentation or instance-level masking to suppress potentially dynamic regions during localization and mapping [3,4]. More recent approaches further improve dynamic-feature handling through stronger segmentation priors, foundation-model-based masks, or detection combined with geometric and temporal consistency cues [7,8,9,10,11,12,13,14,15]. Although these studies demonstrate the effectiveness of semantic awareness in dynamic-scene SLAM, they also highlight an enduring trade-off between robustness and efficiency: segmentation-heavy methods often introduce substantial computational overhead, while detection-driven methods may remain sensitive to boundary instability, missed detections, and delayed suppression of dynamic features.

Besides semantic filtering, the robustness of the feature front-end itself is also critical in dynamic indoor scenes. When motion blur, partial occlusion, or rapid appearance changes occur, handcrafted detectors may yield less stable keypoints, which can weaken data association even after dynamic regions are partially filtered. Learned local features, especially SuperPoint, have shown strong repeatability and robustness under viewpoint and illumination variations and thus provide an attractive option for improving front-end stability [16]. Nevertheless, fully replacing both detector and descriptor in a mature SLAM system may increase computational cost and require substantial redesign of downstream modules, such as binary matching, bag-of-words place recognition, and loop closure. This motivates a hybrid strategy in which the detector is strengthened using learned features, while the efficient ORB descriptor and the mature ORB-SLAM3 backend are preserved [2,17].

**Figure 1:**
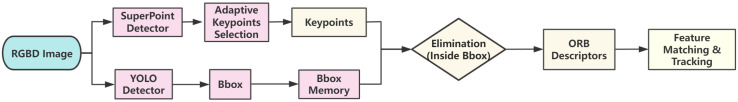
Overview of the SY-SLAM front end. The red modules indicate the components modified or newly introduced in this work relative to the ORB-SLAM3 baseline, whereas the yellow modules represent the retained baseline modules. The arrows indicate the data flow. The SuperPoint and YOLOv8s branches run in parallel, and keypoints inside person bounding boxes are eliminated before ORB descriptor extraction and feature matching.

Motivated by these observations, this paper proposes SY-SLAM, a real-time dynamic RGB-D SLAM system for indoor environments with frequent human motion. The name SY-SLAM reflects the two key front-end components of the proposed system. The letter S stands for SuperPoint, which is used as a detector-only learned keypoint front end, whereas Y stands for YOLO, which provides asynchronous person-aware keypoint suppression based on detected human bounding boxes. Because human motion is the dominant source of dynamic interference in the evaluated indoor RGB-D scenes, we focus on person-aware suppression rather than generic multi-class semantic filtering. The framework nevertheless remains extensible to broader dynamic categories. Built upon the RGB-D configuration of ORB-SLAM3, SY-SLAM retains the original back-end modules, including tracking, local mapping, loop closing, bundle adjustment, and place recognition, while redesigning only the front-end feature extraction and filtering stages. Specifically, we replace the original ORB/FAST detector with a TensorRT-deployed SuperPoint detector while retaining ORB binary descriptors for efficient matching and compatibility [16,17]. To avoid feature starvation after dynamic filtering, we further introduce an adaptive SuperPoint keypoint selection strategy that tightens selection when candidate keypoints are abundant and relaxes the selection constraints when they are scarce. In parallel, an asynchronous YOLOv8s TensorRT thread performs person-only detection with temporal bounding-box memory [18], and keypoints inside detected person regions are removed before ORB descriptor computation and matching, thereby reducing dynamic-feature contamination at the earliest useful stage of the front end. Figure 1 provides an overview of the proposed SY-SLAM front end, highlighting the parallel SuperPoint and YOLOv8s branches and the elimination of person-region keypoints before ORB descriptor extraction and matching.

We evaluate SY-SLAM on five dynamic sequences from the TUM RGB-D fr3 benchmark using absolute trajectory error (ATE) and relative pose error (RPE) [19]. Experimental results show that, compared with ORB-SLAM3, SY-SLAM substantially improves trajectory accuracy in the evaluated highly dynamic walking sequences while maintaining real-time performance. On the four walking sequences, the proposed system reduces ATE RMSE by 93.45% overall, and the complete system consistently outperforms its individual ablation variants, indicating that learned keypoint detection and early dynamic suppression are complementary. In addition, SY-SLAM operates in real time at 46.8 Hz on an Intel i9-13900H CPU with an NVIDIA RTX 4070 Laptop GPU, demonstrating that improved dynamic trajectory accuracy can be achieved without sacrificing practical throughput. Compared with recent dynamic-scene SLAM studies, the proposed method targets a pragmatic design point that balances robustness, compatibility, and runtime efficiency.

The main contributions of this work are fourfold:

1. We propose a detector-only SuperPoint integration for ORB-SLAM3 to improve keypoint repeatability and robustness while preserving ORB-based matching efficiency and back-end compatibility.
2. We introduce an adaptive keypoint selection mechanism to balance keypoint quality and quantity, tightening selection when keypoints are abundant and relaxing it under feature scarcity to maintain stable tracking.
3. We introduce an asynchronous YOLOv8s-based person-aware keypoint suppression module with temporal bounding-box memory, in which keypoints inside detected human regions are removed before descriptor computation and matching.
4. We validate the proposed system through experiments on five dynamic TUM RGB-D fr3 sequences, including quantitative evaluation, ablation analysis, runtime analysis, and qualitative validation in a real indoor environment.

The remainder of this paper is organized as follows. Section 2 reviews related work. Section 3 presents the proposed SY-SLAM framework and implementation details. Section 4 reports the experimental setup and results, including quantitative evaluation, ablation, runtime analysis, and real-world validation. Section 5 discusses comparisons with recent dynamic-scene SLAM methods and the limitations of the proposed system. Section 6 concludes the paper and outlines future work.

## 2. Related Work

### 2.1. Feature-Based RGB-D SLAM Backbones in Practice

**Figure 2:**
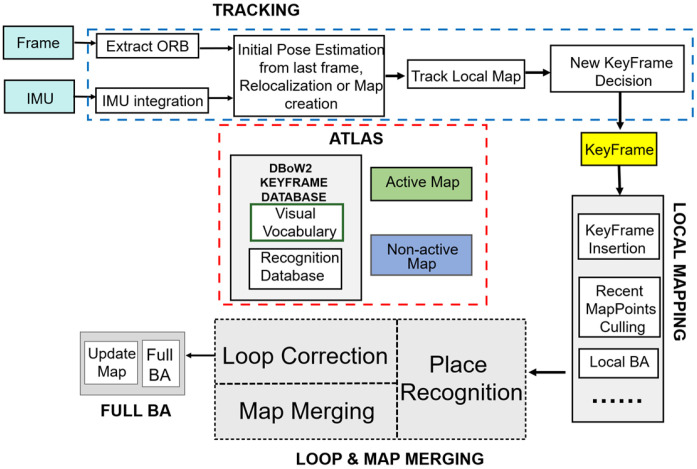
Main system components of ORB-SLAM3, including tracking, local mapping, and loop closing.

Feature-based SLAM remains a dominant paradigm for real-world robotics and indoor mapping due to its favorable accuracy–efficiency trade-off and mature optimization backends. Representative systems such as ORB-SLAM2 and ORB-SLAM3 build keyframe-based maps using sparse keypoints and binary descriptors, supported by robust pose estimation, local bundle adjustment, and loop-closing modules [1,2]. Despite their strong performance in mostly static scenes, their front ends still rely on the implicit rigidity assumption: moving objects can introduce inconsistent correspondences, leading to unstable tracking, biased pose estimates, and map corruption—especially when dynamic regions dominate the image or occlude static structures [3,4]. For reference, Figure 2 summarizes the main system components of ORB-SLAM3, including tracking, local mapping, and loop closing.

### 2.2. Dynamic-Scene SLAM with Semantic Segmentation or Instance Masks

Semantic segmentation and instance-mask-based dynamic SLAM methods aim to suppress moving-object interference at the pixel level and therefore can provide fine-grained dynamic region exclusion in crowded indoor scenes. A representative early system is DynaSLAM, which integrates Mask R-CNN with ORB-SLAM2 and combines semantic cues with multi-view geometry to remove dynamic objects and reconstruct a static map [3]. DS-SLAM follows a related direction by coupling SegNet-based semantic segmentation with motion-consistency checks, improving robustness in dynamic environments while also producing semantic map representations [4]. These methods demonstrated that semantic priors are effective for filtering dynamic regions, but they also revealed the practical cost of dense pixel-level processing in terms of runtime and system complexity.

More recent works further extend this segmentation-driven line. USD-SLAM introduces a large segmentation model (SegGPT) into visual SLAM to improve dynamic-scene robustness [11], while CS-SLAM adopts the lightweight Cross-SegNet architecture together with auxiliary masking strategies to reduce the influence of dynamic objects [14]. In a similar spirit, CA-SLAM combines contour-aware reasoning with RGB-D sensing to improve dynamic-region delineation [13]. Recent SAM-based methods also push this direction further: DZ-SLAM integrates FastSAM with adaptive dense optical flow to handle unknown dynamic elements [13], whereas DN-SLAM combines semantic segmentation, SAM-based priors, and implicit scene modeling to improve localization and reconstruction in dynamic scenes [20]. These studies confirm the potential of strong semantic priors for dynamic-scene SLAM, especially when the dynamic regions are large or geometrically ambiguous.

However, segmentation- and mask-driven pipelines still face several practical limitations. First, dense semantic processing often introduces substantial computational overhead, which can weaken real-time feasibility on consumer hardware. Second, aggressive pixel-level masking may remove valid static background features near dynamic object boundaries, especially in low-texture regions or under coarse mask predictions. Third, many recent systems remain constrained to RGB-D settings, lack open-source implementations, or do not report sufficiently clear runtime analyses, making practical deployment and fair comparison more difficult. These limitations motivate lighter front-end strategies that preserve the key benefit of semantic awareness—namely, dynamic feature suppression—while reducing the cost and feature loss associated with full-frame dense masking.

### 2.3. Detection and Geometry-Based Dynamic Feature Elimination

To achieve a better balance between robustness and real-time performance, another line of work suppresses dynamic features using object detectors together with geometric, motion, or temporal consistency cues, rather than relying solely on full-frame semantic segmentation. Detect-SLAM demonstrates that integrating object detection into the SLAM front end can guide feature selection and reduce dynamic contamination during tracking [7]. RDS-SLAM further introduces semantic tracking and optimization threads to remove dynamic features while preserving practical efficiency, although its computational overhead remains non-negligible [8]. More recently, several approaches have combined one-stage detectors from the YOLO family with optical flow, multi-view verification, or depth cues to identify truly dynamic features more selectively.

Representative examples include FFD-SLAM, which integrates YOLO-based detection and optical flow into an ORB-SLAM2 framework to refine dynamic-feature filtering while preserving real-time capability [14], and the deep-learning-based dynamic SLAM method proposed by Su et al. [21]. These studies suggest that adding a parallel semantic thread and filtering feature points during tracking can improve robustness in highly dynamic scenes. Recent extensions of this direction continue to emphasize efficiency-aware dynamic suppression. DOA-SLAM combines real-time instance segmentation, lightweight object association, and motion-state reasoning to reduce the impact of dynamic objects with limited time cost [12], while DFT-VSLAM integrates YOLOv8s-based object detection with optical-flow masks to jointly eliminate dynamic feature points inside and outside detected object regions [15]. Compared with dense segmentation pipelines, these methods more directly target the feature set used for pose estimation and therefore often provide a more favorable accuracy–efficiency trade-off.

### 2.4. Learned Local Features and Their Integration into SLAM Front-Ends

**Figure 3:**
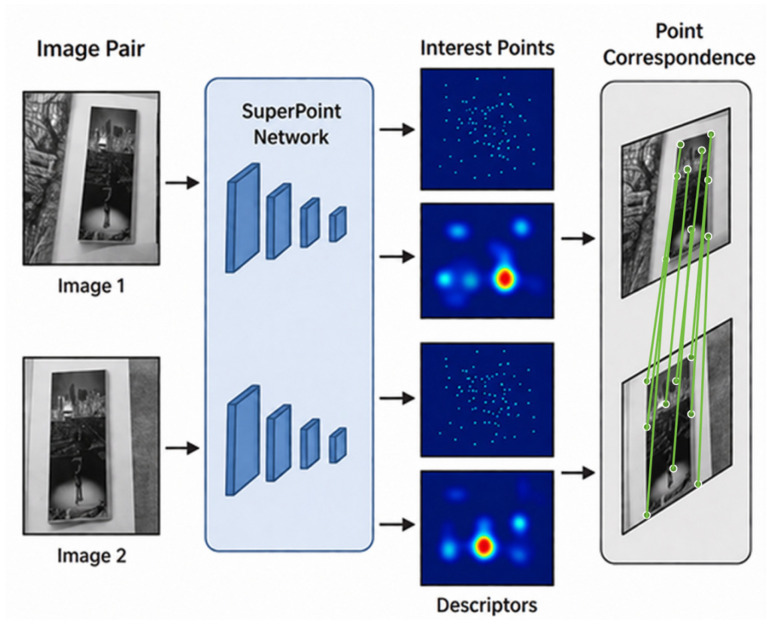
SuperPoint for geometric correspondences, jointly producing interest points and descriptors from image pairs.

Beyond semantic suppression, the robustness of the feature front end itself plays a critical role in dynamic scenes. Learned keypoint detectors such as SuperPoint have shown strong repeatability and robustness under viewpoint and illumination changes, supporting more stable correspondence establishment under motion and partial occlusions [16]. Figure 3 illustrates the SuperPoint framework for geometric correspondences. Motivated by the robustness of learned features, recent systems explore replacing handcrafted keypoints/descriptors in classic pipelines. SuperPoint-SLAM3 augments ORB-SLAM3 with deep features and proposes adaptive keypoint distribution (adaptive NMS) and learning-based loop closure to improve localization and mapping fidelity under challenging visual conditions [22]. In parallel, learning-based SLAM systems and deep matching advances—e.g., DROID-SLAM for deep visual SLAM and LightGlue for fast local feature matching—indicate the ongoing shift toward learned representations for robustness [23,24].

Nevertheless, fully replacing both detector and descriptor, or redesigning loop closure and map representations, can increase computational cost and integration burden. This has motivated hybrid strategies that preserve efficient binary descriptor matching while selectively introducing learned components to improve repeatability and stability at the detection stage—especially when real-time performance must be maintained.

### 2.5. Positioning with Respect to This Work

In summary, prior dynamic SLAM research broadly follows (i) segmentation-driven suppression, (ii) detection + geometric-consistency-based elimination, and (iii) learned-feature-driven front ends. Each direction offers robustness benefits but may trade off runtime, complexity, or compatibility. Accordingly, this work focuses on a detector-only learned front end combined with early keypoint-level dynamic suppression, rather than a full deep-feature replacement of the original ORB-SLAM3 pipeline.

## 3. Methodology

### 3.1. System Overview

**Figure 4:**
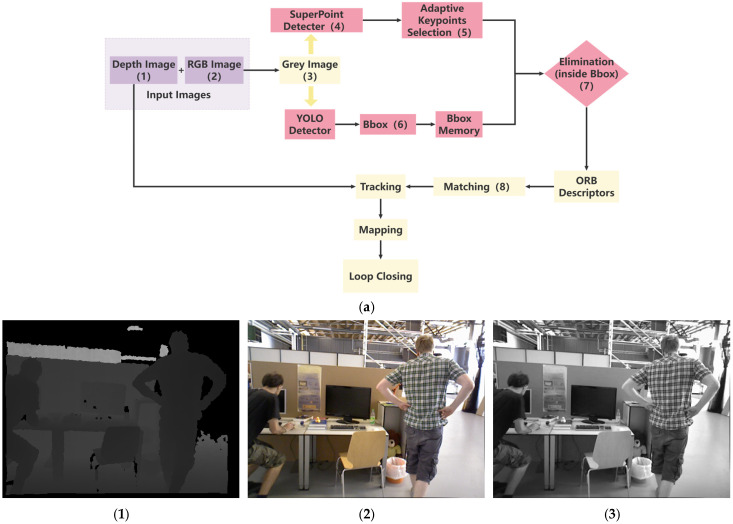

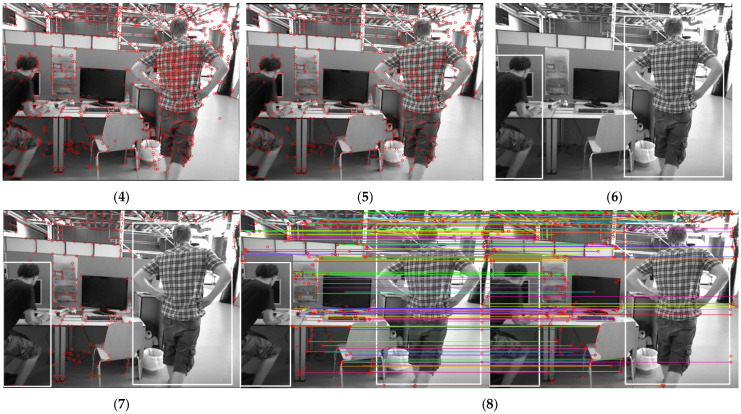
Detailed overview of the SY-SLAM front end integrated into ORB-SLAM3. (**a**) The overall processing pipeline of the proposed SY-SLAM front end. The numbered examples correspond to the numbered modules in the pipeline: (**1**) depth image; (**2**) RGB image; (**3**) grayscale image converted from the RGB image; (**4**) SuperPoint keypoint detection result; (**5**) adaptive keypoint selection result; (**6**) YOLOv8s-based person bounding-box detection result; (**7**) keypoint suppression inside detected person bounding boxes; and (**8**) feature matching result after dynamic-keypoint suppression.

SY-SLAM is built upon the RGB-D configuration of ORB-SLAM3 [2]. The original back end, including tracking, local mapping, loop closing, bundle adjustment, and place recognition, is preserved, while the front end is redesigned to improve trajectory accuracy in dynamic indoor scenes with frequent human motion. As shown in Figure 4, the RGB and depth images are first fed into the system. The RGB image is converted to grayscale for front-end processing, and the grayscale branch is then divided into two parallel paths. In the first path, a TensorRT-deployed SuperPoint detector-only module extracts candidate keypoints, which are further refined by an adaptive keypoint selection step to balance keypoint quality and quantity. In the second path, an asynchronous YOLOv8s detector performs person-only detection, and the predicted bounding boxes are temporally stabilized through a bbox memory module.

The outputs of these two paths are fused before feature description. Specifically, keypoints falling inside the predicted person regions are removed by the bounding-box elimination module, so that dynamic features are suppressed before they enter descriptor extraction and matching. ORB binary descriptors are then computed only for the remaining keypoints, preserving full compatibility with ORB-SLAM3’s existing matching and place-recognition pipeline. After this front-end filtering, the resulting features are used for matching and pose tracking, while the RGB-D depth information is retained for metric reconstruction and the original ORB-SLAM3 back-end handles mapping and loop closure. In this way, SY-SLAM combines learned keypoint detection, adaptive keypoint selection, and temporally stabilized person-aware suppression within a unified front end, while maintaining the efficiency and maturity of the ORB-SLAM3 framework.

### 3.2. SuperPoint Detector-Only Integration with ORB-SLAM3

#### 3.2.1. Motivation for Integrating Learned Detection into ORB-SLAM3

**Figure 5:**
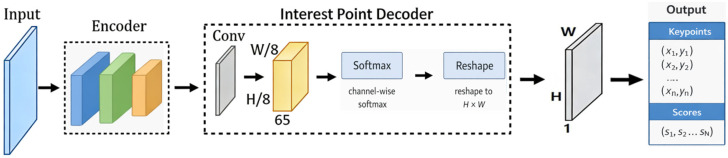
SuperPoint detector head for interest point prediction, producing keypoint locations and confidence scores.

ORB-SLAM3 relies on ORB features for tracking, mapping, and bag-of-words (BoW) place recognition [2]. While ORB is efficient, handcrafted detectors may become unstable when the scene contains motion blur, partial occlusions, or rapid appearance changes. SuperPoint is a learned interest point detector with strong repeatability across viewpoint and illumination variations [16]. Figure 5 shows the detector head of SuperPoint, which outputs keypoint locations together with their confidence scores.

Instead of replacing the entire feature pipeline, SY-SLAM adopts a detector-only strategy: SuperPoint provides keypoint locations and confidence scores, while we retain the standard ORB binary descriptor [17] computed at the detected keypoints. This design preserves ORB-SLAM3’s matching efficiency and compatibility with its downstream modules (e.g., BoW-based loop closing), while improving the stability of the detected keypoints.

#### 3.2.2. Multi-Scale Keypoint Detection and Spatial Distribution

ORB-SLAM3 builds an image pyramid $\{I_{t}^{l}{\}}_{l=0}^{L-1}$ to improve scale robustness. SY-SLAM follows the same pyramid construction and performs SuperPoint detection at pyramid levels to align with the existing ORB-SLAM3 feature organization. In our implementation, SuperPoint is executed independently at each pyramid level rather than being run once on the original image and then mapped to all levels. For each level l, we perform the following operations. The pyramid image $I_{t}^{l}$ is resized to the SuperPoint network input resolution (640 × 480 in our implementation), and SuperPoint inference is performed on that resized level image. SuperPoint inference outputs a set of candidate keypoints, denoted by ${\tilde{K}}_{t}^{l}$ in Equation (1):

(1) $${\tilde{K}}_{t}^{l}=\{(x_{i}^{l},y_{i}^{l},s_{i}^{l}){\}}_{i=1}^{N_{l}},$$

where $\left(x_{i}^{l},y_{i}^{l}\right)$ denote 2D coordinates rescaled back to pyramid level l from the SuperPoint network input and $s_{i}^{l}\;$ is the detector confidence. An adaptive keypoint selection strategy is applied to avoid excessive clustering and improve spatial coverage. Then we follow ORB-SLAM’s octree-based distribution strategy to select the final keypoints per level. Specifically, we keep a large candidate pool and then sample a fixed per-level budget using octree partitioning, matching the feature allocation policy used in ORB-SLAM3. SuperPoint confidence $s_{i}^{l}$ is stored as the keypoint response to guide selection.

#### 3.2.3. ORB Descriptors on SuperPoint Keypoints

Given the final set of keypoints ${\;K}_{t}^{l}$ at each pyramid level, we compute keypoint orientation using the ORB orientation procedure and ORB descriptors (256-bit, i.e., 32 bytes per keypoint) at the selected locations [17]. As a result, the output feature representation $(K_{t},D_{t})\;$ is fully compatible with ORB-SLAM3’s matching, tracking, mapping, and loop closing.

### 3.3. Asynchronous YOLOv8s Dynamic Detection and Keypoint Suppression

#### 3.3.1. Design Rationale

Dynamic human motion is one of the dominant sources of outliers in indoor RGB-D SLAM: features on moving people can generate incorrect correspondences, causing drift, tracking loss, and map corruption. Dynamic SLAM systems commonly reduce such effects by identifying dynamic regions (e.g., via semantic segmentation or detection) and excluding them from geometric estimation [3,4,25]. Accordingly, in this work, the YOLO branch is used for bounding-box-based keypoint suppression rather than pixel-level semantic masking. We treat the person category as the primary dynamic class and remove keypoints falling inside detected person bounding boxes before ORB descriptor computation and matching. This design targets the dominant source of dynamic disturbance in indoor human-populated scenes while keeping the front end lightweight and real-time.

#### 3.3.2. Asynchronous Detection Thread

To avoid slowing down tracking, YOLOv8s [18] runs in a separate thread. Let $B_{t}$ denote the set of detected person bounding boxes available to the tracking thread at time t. Each incoming RGB frame is pushed to the detector thread for asynchronous inference, while only the most recent pending image is retained in the detector queue to reduce latency accumulation. The tracking thread does not wait for detector completion; instead, at each tracking step, it queries the most recent available detection result. If a new result is available, $B_{t}$ is updated accordingly; otherwise, the last cached result is reused. The resulting stabilized bounding boxes are then fed into the feature extractor before frame construction. In this way, perception latency is decoupled from front-end tracking, while the detector output remains temporally aligned with recent frames as closely as possible.

#### 3.3.3. Bounding-Box Memory for Temporal Stability

Object detectors may occasionally miss a person region due to motion blur, partial occlusion, or viewpoint changes. To reduce such temporal flicker, SY-SLAM maintains a short-term memory of detected person bounding boxes. In implementation, only detections of the COCO person class with a confidence threshold of 0.5 are retained. Each valid box is clamped to the image boundary and expanded by a fixed 2-pixel margin before filtering. If a new detector output contains at least one valid person box, the stored box set is updated immediately. If a new detector output is available but contains no valid person box, the previous box set is retained for up to three consecutive empty updates before being cleared. The resulting temporally stabilized bounding boxes are then fed into the feature extractor before frame construction, so that dynamic-region keypoint suppression remains stable under short-term detector misses.

#### 3.3.4. Keypoint-Level Elimination Inside Dynamic Regions

Given the selected keypoints $\;K_{t}^{l}\;$ extracted at each pyramid level l, we map their coordinates back to the original image coordinate system according to Equation (2):

(2) $$p_{i}=\alpha_{l}\;p_{i}^{l},$$

where $\alpha_{l}$ denotes the scale factor from level l to the original resolution. We then eliminate keypoints whose mapped locations fall inside any detected dynamic bounding box (optionally expanded by a small margin to account for boundary jitter). The resulting static keypoint set is defined in Equation (3):

(3) $$K_{t,static}^{l}=\{k_{i}\in K_{t}^{l}|p_{i}\notin {⋃}_{b\in B_{t}}(b⊕\delta )\},$$

**Figure 6:**
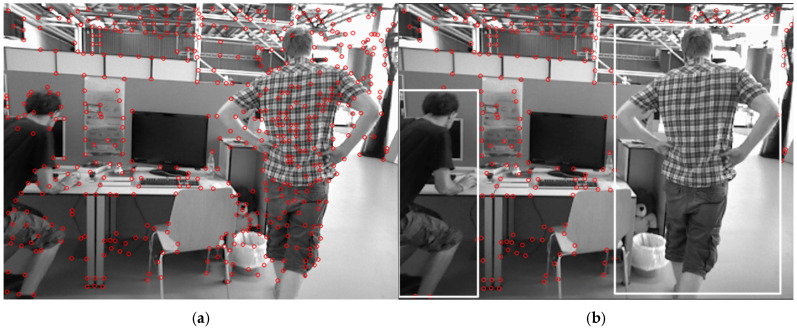
Visualization of dynamic-region keypoint suppression: (**a**) raw keypoints before suppression and (**b**) retained keypoints after person-region suppression.

Here, ⊕δ denotes box expansion by a small margin δ. Importantly, this elimination is performed before ORB descriptor computation and matching. Thus, dynamic keypoints never contribute descriptors, correspondences, or map points, reducing both outlier ratio and unnecessary computation. Figure 6 visualizes this dynamic-region keypoint suppression process, showing the keypoints before and after person-region filtering.

### 3.4. Adaptive SuperPoint Keypoint Selection Under Feature Scarcity

#### 3.4.1. Problem Setting

While suppressing dynamic regions improves robustness, it may also reduce the number of usable keypoints, especially in low-texture areas, blurred frames, and frames with large dynamic coverage where many features are intentionally removed. Insufficient keypoints can cause unstable pose estimation and tracking failure. Therefore, SY-SLAM introduces an adaptive SuperPoint-only keypoint selection mechanism to maintain a minimum keypoint budget without introducing additional detectors. When candidates are abundant, we apply stricter filtering to prioritize higher-quality keypoints.

#### 3.4.2. Adaptive Relaxation of SuperPoint Selection

Let $n_{l}\;$ be the desired per-level keypoint budget, following ORB-SLAM3’s allocation across pyramid levels. For each pyramid level l, SuperPoint first produces a candidate set ${\tilde{K}}_{t}^{l}$. In our implementation, the base configuration uses a SuperPoint input resolution of 640×480, an initial confidence threshold of 0.004, and an ANMS radius of 4. The adaptive selection then proceeds in three stages. First, candidate keypoints are extracted under the default configuration. Second, if the candidate count satisfies $|{\tilde{K}}_{t}^{l}|\;<0.6\;n_{l}$, the confidence threshold is progressively relaxed from 0.004 by multiplying it by 0.5 at each round, for up to three rounds, so that weaker but still potentially useful keypoints can be retained. Third, if the candidate count remains below $|{\tilde{K}}_{t}^{l}|\;<0.2\;n_{l}$, the ANMS radius is progressively relaxed from 4 to 2 and then to 1, for up to three rounds, in order to preserve more candidates in sparse-image regions. Finally, instead of immediately truncating the candidates to the final per-level target $n_{l}$, we retain a larger candidate pool before octree redistribution, with the pool size set to $\min\left(1500,5n_{l}\right)$ per level and then apply the original ORB-SLAM3 octree-based allocation strategy to obtain the final per-level keypoints with improved spatial coverage.

**Figure 7:**
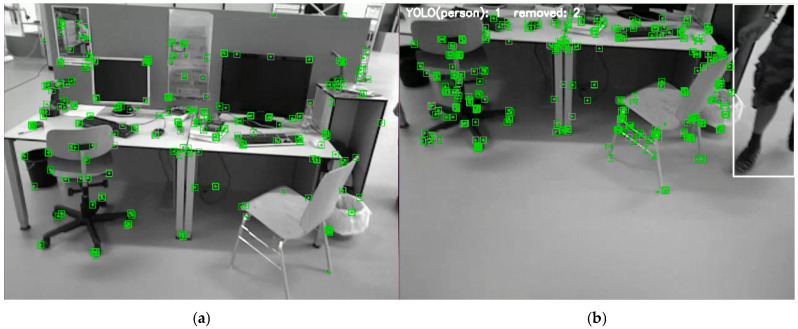
Adaptive SuperPoint keypoint selection under feature scarcity: (**a**) sufficient keypoints under normal conditions and (**b**) increased keypoint yield after adaptive relaxation in a feature-scarce frame.

After the relaxation steps, we apply the same octree-based distribution strategy so that the final per-level keypoints follow the ORB-SLAM3 allocation policy while preserving spatial coverage. This adaptive mechanism is activated only when needed; under normal conditions, the default SuperPoint selection provides sufficient stable features. Figure 7 illustrates the effect of adaptive SuperPoint keypoint selection under feature scarcity by comparing the default selection under normal conditions with the relaxed selection after adaptive relaxation.

### 3.5. Integration into ORB-SLAM3 and Implementation Notes

SY-SLAM modifies only the feature extraction and filtering stages of ORB-SLAM3. All back-end modules—including RGB-D depth association, local mapping, loop closing, and bundle adjustment—remain unchanged [2]. By retaining ORB descriptors [17], SY-SLAM preserves ORB-SLAM3’s efficient matching and compatibility with its existing data structures and place recognition pipeline. For real-time performance, SuperPoint and YOLOv8s inference are accelerated with TensorRT.

## 4. Experimental Results

### 4.1. Implementation Details and Hardware

The learned keypoint detector of SY-SLAM is deployed as a TensorRT-accelerated SuperPoint [26] model with pre-trained weights. In our implementation, SuperPointTRT is enabled and configured with an input resolution of 640 × 480, which is the same as that of the TUM RGB-D dataset. During feature extraction, SuperPoint inference is performed separately on every pyramid level. To encourage a well-distributed keypoint set while preserving sufficient candidates for downstream selection, we apply NMS with a radius of 4, keep up to 1500 candidates per pyramid level, and use a confidence threshold of 0.004.

For dynamic human suppression, YOLOv8s is deployed as a TensorRT engine [27] in an asynchronous thread. In our implementation, the detector follows the end-to-end YOLOv8-TensorRT deployment pipeline, in which bounding-box decoding and NMS are built into the model/engine. The YOLO inference input size is 640 × 640, and the TensorRT engine is built in FP16 mode for half-precision inference. During engine export/building, the NMS IoU threshold is set to 0.65. At the SLAM front-end level, we further retain only the COCO person class and apply a person-box confidence threshold of 0.5 before keypoint suppression. The detector is executed at every frame; however, because inference runs asynchronously, tracking is not blocked by detection latency. The predicted person bounding boxes are temporally stabilized with a lightweight bounding-box memory to reduce flickering, and a fixed margin of 2 pixels is added to each bounding-box boundary before filtering.

All experiments are conducted on a laptop platform equipped with a 13th Gen Intel(R) Core(TM) i9-13900H CPU, an NVIDIA GeForce RTX 4070 Laptop GPU, and 32 GB RAM, compiled and run on Ubuntu 22.04 LTS operating system.

### 4.2. Datasets and Evaluation Metrics

**Figure 8:**
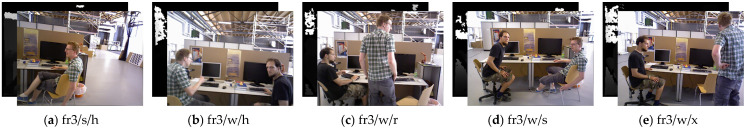
Representative frames from the five evaluated TUM RGB-D fr3 sequences: (**a**) fr3/s/h, (**b**) fr3/w/h, (**c**) fr3/w/r, (**d**) fr3/w/s, and (**e**) fr3/w/x.

To evaluate the performance of SY-SLAM in dynamic indoor environments, experiments are conducted on the TUM RGB-D dataset [19], which was collected by Technische Universität München (TUM), Germany. The dataset provides synchronized RGB-D streams together with high-precision motion-capture ground-truth trajectories for quantitative evaluation. In particular, we focus on sequences from the fr3 subset containing representative indoor scenes with people walking or sitting, where the static-world assumption is frequently violated and dynamic objects may introduce feature contamination that leads to tracking drift or failure, as also discussed in previous dynamic SLAM studies. Figure 8 shows representative frames from the five evaluated TUM RGB-D fr3 sequences.

Following this motivation, we report results on five sequences that cover different camera motions and levels of dynamic interference: fr3/walking_xyz, fr3/walking_rpy, fr3/walking_static, fr3/walking_halfsphere, and fr3/sitting_halfsphere. For concise presentation in the result tables, we denote them as fr3/w/x, fr3/w/r, fr3/w/s, fr3/w/h, and fr3/s/h, respectively. Since RGB-D provides metric scale, all trajectories are evaluated in the original scale without scale correction.

For quantitative evaluation, we compute trajectory errors using the evo toolkit [28] and report both global accuracy and local drift, consistent with standard practice in RGB-D SLAM benchmarking. Specifically, we evaluate the absolute trajectory error (ATE) and the relative pose error (RPE). Metric ATE stands for global consistency of trajectory, while metric RPE measures the translational and rotational drift. We present the values of RMSE, mean error, median error, and standard deviation (S.D.) in this paper. We also show the values of improvement of SY-SLAM compared to the original ORB-SLAM3. The improvement rate is defined in Equation (4):

(4) $$Improvement=\frac{n-m}{n}\times 100\%\;,$$

where *m* and n represent the corresponding metric values of the improved method and ORB-SLAM3, respectively. Unless explicitly stated otherwise, the benchmark results reported correspond to single representative runs under fixed parameter settings, and the resulting trajectories are evaluated against ground truth using the same toolchain and alignment settings.

### 4.3. Quantitative Accuracy Results on TUM fr3 Dynamic Sequences

Table 1 summarizes the absolute trajectory error (ATE) on the five dynamic TUM RGB-D fr3 sequences. Overall, SY-SLAM yields substantially lower ATE than the ORB-SLAM3 baseline, indicating improved global trajectory consistency under dynamic interference. The most significant gains are observed on the four walking sequences, where moving humans occupy a larger portion of the scene and more strongly violate the static-world assumption. In particular, the ATE RMSE decreases from 0.4194 m to 0.0160 m on fr3/w/x, from 0.7018 m to 0.0456 m on fr3/w/r, from 0.1528 m to 0.0068 m on fr3/w/s, and from 0.2614 m to 0.0299 m on fr3/w/h. The particularly pronounced improvement on fr3/w/s (i.e., fr3/walking_static) can be explained by the motion characteristics of this sequence, where the camera remains essentially static while the main disturbance comes from people walking through the scene, so suppressing person-induced dynamic outliers before descriptor computation and matching yields an especially large gain in pose estimation accuracy. By contrast, the improvement on fr3/s/h is smaller (38.22%), which is reasonable because this sequence is relatively less affected by severe dynamic interference and the ORB-SLAM3 baseline already performs strongly. This is also consistent with the fact that ORB-SLAM3 includes generic robust geometric estimation and outlier rejection, which can tolerate a limited amount of dynamic-induced mismatch in mildly dynamic cases, even though it is not a dedicated dynamic-object filtering system. Overall, the ATE results confirm that the proposed front-end is particularly effective when dynamic objects dominate the image and introduce large numbers of unstable correspondences. To further evaluate short-term drift behavior, Table 2 and Table 3 report the relative pose error (RPE) in translation and rotation, respectively. SY-SLAM consistently reduces both translational and rotational RPE on the more challenging dynamic sequences, showing that the proposed front end not only improves global accuracy but also stabilizes local frame-to-frame motion estimation. For example, on fr3/w/s, the translation RPE RMSE decreases from 0.0147 m to 0.0066 m, while the rotation RPE RMSE decreases from 0.2988° to 0.1757°. Similarly, on fr3/w/x, the translation RPE RMSE decreases from 0.0228 m to 0.0123 m, and the rotation RPE RMSE decreases from 0.5371° to 0.3967°. These improvements indicate that the combination of learned keypoint detection and early dynamic-region suppression helps maintain more reliable correspondences between adjacent frames. The gains are more modest on fr3/s/h, where both translation and rotation errors are already small for the baseline, again suggesting that the proposed method is most beneficial in sequences with stronger dynamic disturbances.

**Figure 9:**
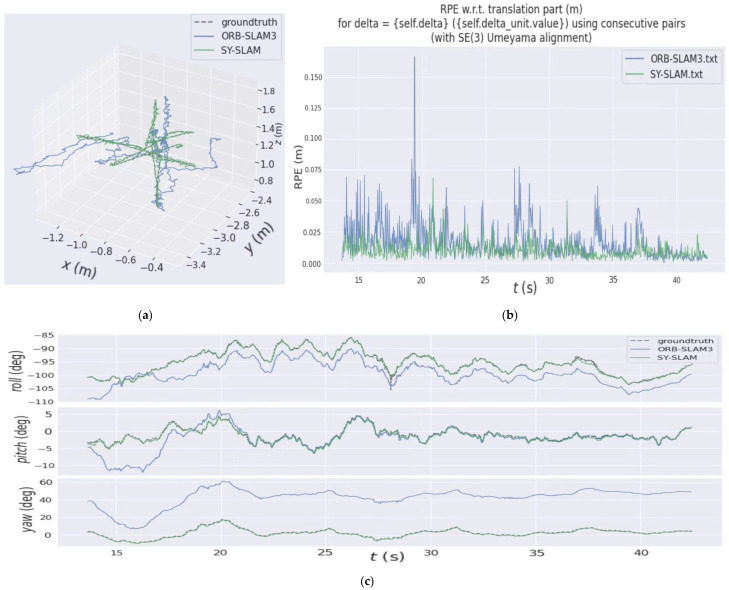
Quantitative comparison between ORB-SLAM3 and SY-SLAM on the fr3/w/x sequence: (**a**) 3D trajectory comparison, (**b**) translational RPE comparison, and (**c**) roll, pitch, and yaw comparisons.

The visualizations in Figure 9 are consistent with the quantitative results. Using the representative fr3/w/x sequence as an example, the figure compares the trajectories and error profiles of ORB-SLAM3 and SY-SLAM under strong dynamic interference. In this sequence, ORB-SLAM3 shows larger trajectory deviations and more pronounced error fluctuations, whereas SY-SLAM maintains a trajectory that is more closely aligned with the ground truth and exhibits a visibly smoother error profile. This behavior can be explained by the proposed front-end design. The YOLO-based dynamic-region suppression reduces the number of dynamic keypoints entering descriptor extraction and matching, thereby lowering the chance of mismatch propagation into pose estimation. At the same time, the detector-only SuperPoint front end improves the repeatability of the remaining static keypoints, while the adaptive keypoint selection strategy helps maintain sufficient feature support even after dynamic-region filtering. As a result, SY-SLAM achieves both lower trajectory drift and more stable short-term motion estimation in dynamic indoor scenes.

### 4.4. Ablation Study

To isolate the contribution of each proposed component, we evaluate four variants: the original ORB-SLAM3 front end, SuperPoint-only (replacing the detector while keeping ORB descriptors), ORB + YOLO (dynamic-region suppression while retaining ORB detection), and SuperPoint + YOLO (the full SY-SLAM front end).

The results in Table 4 show that dynamic-region suppression is the primary source of improvement in the highly dynamic walking sequences, where ORB + YOLO already reduces the ATE RMSE by a large margin compared with ORB-SLAM3. However, this gain is not uniform across all sequences. On fr3/s/h, for example, ORB + YOLO is slightly worse than the ORB-SLAM3 baseline, suggesting that dynamic suppression alone may introduce overfiltering when the dynamic interference is relatively limited. By contrast, SuperPoint-only provides sequence-dependent gains, particularly in sequences where repeatability and robustness of the retained static features are critical. Importantly, the full SuperPoint + YOLO configuration consistently achieves the best overall results, indicating that learned keypoint detection and dynamic-region suppression are complementary: the latter removes dynamic outliers before matching, while the former improves the stability and quality of the remaining correspondences.

### 4.5. Runtime Analysis

Real-time performance is one of the key design objectives of SY-SLAM. To ensure a fair comparison, SY-SLAM and ORB-SLAM3 were executed on the same computing platform, and their computation times on the fr3/w/x sequence were compared. The results are summarized in Table 5. SY-SLAM achieves an end-to-end wall-clock time of 21.36 ms per frame (≈46.8 Hz), indicating that the proposed system satisfies the real-time requirement of RGB-D SLAM in dynamic indoor scenes. The runtime breakdown further shows that the tracking thread costs 11.04 ms on average, while the asynchronous YOLO thread costs 2.66 ms. For the reported runtime in Table 5, the asynchronous detection branch uses the above TensorRT-deployed YOLOv8s configuration, namely 640 × 640 input, FP16 engine mode, built-in end-to-end NMS with an IoU threshold of 0.65, and a front-end person-box confidence threshold of 0.5. This efficiency comes from two complementary design choices: first, the front-end feature extraction is accelerated by a TensorRT-deployed SuperPoint detector-only module, which enables stable keypoint detection at real-time speed and keeps the tracking pipeline lightweight; second, dynamic suppression relies on a TensorRT-accelerated YOLOv8s dynamic detector executed asynchronously, so that detection latency is largely decoupled from tracking and does not dominate the end-to-end processing time. Overall, these results show that SY-SLAM improves trajectory accuracy and front-end stability in dynamic indoor scenes without sacrificing real-time throughput.

### 4.6. Qualitative Validation in a Real-World Environment

**Figure 10:**
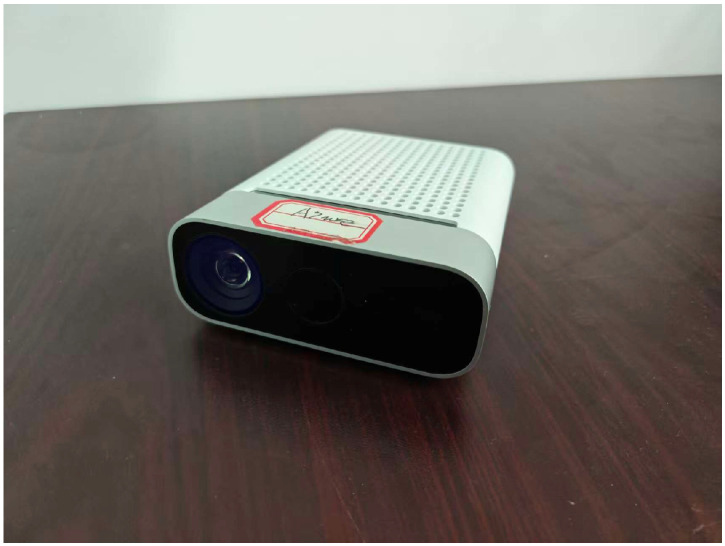
Azure Kinect DK RGB-D camera used in the real-world indoor experiments.

**Figure 11:**
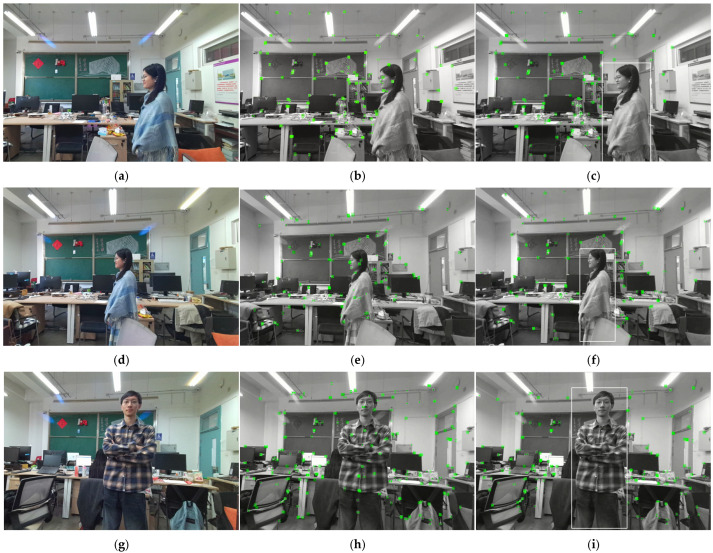
Qualitative comparison between ORB-SLAM3 and the proposed SY-SLAM in a real laboratory environment. Specifically, subfigures (**a**,**d**,**g**) show the RGB images, (**b**,**e**,**h**) show the results of ORB-SLAM3, and (**c**,**f**,**i**) show the results of the proposed SY-SLAM. The three rows correspond to representative scenes captured from different viewpoints. The green dots denote the extracted feature points.

To further validate the practical applicability of the proposed SY-SLAM beyond benchmark datasets, we conducted qualitative experiments in a real laboratory environment using an Azure Kinect DK RGB-D camera (Microsoft Corporation, Redmond, WA, USA). Because no external motion-capture system or other ground-truth localization device was available in our laboratory setup, this real-world experiment is intended as a qualitative validation of practical applicability rather than a fully quantitative benchmark with ATE/RPE reporting. Figure 10 shows the camera used for data collection, and Table 6 summarizes the main camera and front-end settings adopted in the real-world experiments. The intrinsic parameters listed in Table 6 are the factory-default values provided by the Azure Kinect DK, and no dedicated camera calibration was performed prior to the experiment. In addition, unlike the TUM benchmark experiments, which used 640 × 480 input images, the real-world Azure Kinect experiments were conducted at 1280 × 720 resolution. This higher input resolution increases the image size and may slightly increase the runtime of the tracking and asynchronous YOLO branches. At the same time, the higher resolution preserves richer visual details and generally provides clearer scene structures and more discriminative features, which is favorable for improving tracking accuracy in practice. Therefore, this real-world experiment should be interpreted as a qualitative validation under a different camera setting, rather than as a directly controlled runtime comparison with the TUM-based benchmark experiments. Figure 11 presents three representative examples captured from different viewpoints. From left to right, the three columns show the input RGB image, the feature distribution produced by ORB-SLAM3, and that produced by the proposed SY-SLAM, respectively. The green dots denote the retained keypoints used for feature matching.

As shown in Figure 11, when a person moves within the scene, ORB-SLAM3 still preserves a considerable number of keypoints on the dynamic human region, which increases the risk of dynamic-feature contamination and may degrade tracking accuracy. In contrast, the proposed SY-SLAM suppresses most keypoints located on the moving person while preserving sufficient reliable keypoints in static background regions such as desks, walls, doors, and other indoor structures. These qualitative results indicate that SY-SLAM can better separate dynamic and static regions in real indoor environments, thereby improving front-end stability under dynamic interference. For a basic quantitative proxy that does not require external ground-truth measurement, we further counted tracking-loss events on the self-collected real-world sequences. Two RGB-D sequences were recorded in the laboratory environment, containing 197 frames in total, corresponding to approximately 6.57 s at 30 Hz. In this work, a tracking-loss event is defined as a case in which the tracking process enters a lost state and cannot continue normal pose estimation without relocalization or reinitialization. Under this criterion, SY-SLAM maintained continuous tracking on both self-collected sequences, with 0 tracking-loss events out of 197 frames, corresponding to a tracking-failure rate of 0.0% and a tracking-continuity rate of 100%. This proxy metric does not replace trajectory-based ATE/RPE evaluation with external ground truth, but it provides an operational indication of tracking continuity in the self-collected real-world validation. A more rigorous quantitative real-world evaluation with external ground-truth measurement will be included in future work.

## 5. Discussion

### 5.1. Comparison with Dynamic SLAM Baselines

To further position SY-SLAM within recent dynamic-scene SLAM research, we compare it with several representative methods reported in the literature. Since most of these systems were evaluated on different hardware platforms and under different software configurations and many are not fully open source, a strictly controlled comparison under identical conditions is not possible. Table 7 should be interpreted as a literature-level reference for positioning rather than as a direct empirical ranking. As in many recent dynamic-SLAM studies, we use the ATE RMSE on the TUM fr3/w/x sequence as the main comparison metric, since this sequence is widely reported and provides a representative evaluation scenario for dynamic indoor RGB-D SLAM.

As shown in Table 7, several recent methods achieve strong absolute accuracy through semantic segmentation or instance-level masking. For example, DynaSLAM [3] reports a low ATE RMSE by relying on Mask R-CNN to remove dynamic objects, but its dense instance-segmentation pipeline incurs substantial computational overhead and is generally unsuitable for real-time operation. Similarly, SEG-SLAM [29], CS-SLAM [10], and USD-SLAM [9] improve robustness through semantic segmentation or large segmentation models, but their reported tracking-time analyses are either unavailable or indicate limited real-time capability. These methods demonstrate the effectiveness of strong semantic priors, but they also highlight the cost of dense pixel-level processing.

A second group of methods adopts lighter detection-based or hybrid strategies. YOLOv9S-SLAM [30], DFT-VSLAM [15], CA-SLAM [11], YOLOv5 + Clustering [32], and ORB-SLAM + YOLOv5 [33] combine object detection with geometric reasoning, optical-flow masks, contour cues, or clustering-based filtering to reduce the impact of dynamic features. Compared with dense segmentation pipelines, these methods often achieve a better balance between accuracy and runtime. However, many of them still do not provide sufficiently clear tracking-time analyses, making it difficult to judge their practical real-time feasibility. The recently proposed YOLO11-ORB-SLAM3 [31] also follows a lightweight detection-driven direction and reports real-time capability in some cases, but its ATE RMSE on fr3/w/x remains slightly higher than that of SY-SLAM.

From a methodological perspective, SY-SLAM differs from existing hybrid SLAM approaches in that it does not aim to fully replace the ORB-SLAM3 feature pipeline with deep features. Instead, it adopts a detector-only SuperPoint front end while retaining ORB descriptors, ORB-SLAM3 matching, and the original place-recognition and back-end modules. In addition, unlike segmentation-heavy methods or hybrid methods that apply filtering after feature description, SY-SLAM performs early person-aware keypoint suppression before ORB descriptor computation and matching. Together with asynchronous YOLOv8s detection and short-term bounding-box stabilization, this design targets a practical balance among trajectory accuracy, compatibility, and real-time feasibility in dynamic indoor RGB-D scenes.

Within this literature-level comparison, SY-SLAM achieves an ATE RMSE of 0.0160 m on fr3/w/x, which is competitive with the best recent methods and lower than those reported for YOLO11-ORB-SLAM3 (0.0174 m), YOLOv5 + Clustering (0.0174 m), DS-SLAM (0.0247 m), and USD-SLAM (0.0350 m). Although a few methods, such as DynaSLAM and YOLOv9S-SLAM, report slightly lower ATE RMSE values, SY-SLAM retains a distinct advantage in terms of transparent runtime reporting and practical real-time feasibility, since its tracking thread, detection thread, and end-to-end wall-clock time are explicitly reported. This reflects the central design choice of SY-SLAM: instead of relying on dense segmentation, it performs early person-aware keypoint suppression together with a detector-only SuperPoint front end, thereby improving robustness while maintaining real-time throughput.

Overall, the comparison suggests that current dynamic SLAM methods still face a trade-off between localization accuracy, semantic precision, and computational cost. SY-SLAM occupies a practical design point within this landscape: it does not pursue the heaviest semantic modeling, but instead combines lightweight detection-based suppression with learned keypoint repeatability to achieve a favorable balance between accuracy, robustness, and efficiency in dynamic indoor RGB-D scenes.

### 5.2. Limitations

Although SY-SLAM demonstrates improved trajectory accuracy in dynamic environments while maintaining real-time performance, several limitations remain.

Firstly, the current implementation focuses on filtering a single dynamic category, namely persons, because human motion is the dominant source of dynamic interference in the TUM RGB-D sequences considered in this work. While this design is effective in many indoor scenes, other moving objects that do not belong to the modeled class may still introduce dynamic features into the pipeline. As a result, the current system may generalize less well to scenes containing other dynamic objects, such as service robots, carts, chairs, or carried items, which are not explicitly modeled by the current person-only detector. Extending the same suppression framework to multi-class dynamic object filtering is therefore an important direction for future work. A straightforward extension is to generalize the current person-only YOLO branch to a configurable set of dynamic classes defined in the detector label list (e.g., the COCO label set), aggregate their detected bounding boxes, and apply the same pre-descriptor keypoint suppression strategy before ORB descriptor computation and matching. For dynamic categories not covered by the default detector label set, the same framework could be further extended by fine-tuning or retraining the YOLO detector on task-specific datasets and regenerating the deployment engine.

Secondly, the proposed suppression mechanism is based on bounding-box-level filtering rather than refined pixel-level masks. As a result, useful static background features near object boundaries may also be removed together with dynamic ones. This effect can become more pronounced when a person occupies a large portion of the image or in strongly dynamic scenes where the visible static background is limited, in which case tracking accuracy may decrease temporarily. Although the adaptive keypoint selection strategy helps alleviate feature scarcity after suppression, future integration of instance segmentation may better preserve static background features near object boundaries while still suppressing dynamic regions.

Thirdly, the current system does not explicitly verify whether a detected object is actually moving. Consequently, objects belonging to dynamic categories may still be filtered even when they are temporarily stationary. In particular, if a person remains stationary for an extended period, the current person-aware suppression strategy may still remove potentially useful features unnecessarily, thereby reducing feature availability without corresponding dynamic benefit. In future work, this limitation could be alleviated by incorporating additional motion validation mechanisms, such as optical-flow consistency or depth-based geometric checks, which may not only help distinguish truly dynamic objects from merely detectable objects, but also partially compensate for occasional detector misses by providing complementary motion cues.

In addition, the benchmark results reported in Table 1, Table 2 and Table 3 are based on single representative runs rather than repeated-run statistics across independent executions. Future work will include repeated-run evaluation and more comprehensive supplementary reporting to better quantify execution-level variability.

Finally, the evaluation presented here is mainly based on public RGB-D benchmarks and selected recent literature results, many of which were obtained on different hardware platforms and under different software settings. Therefore, the comparison with recent methods should be interpreted as indicative rather than strictly controlled. In addition, the current real-world validation is mainly qualitative, since no external ground-truth measurement system was available in our laboratory setup for the Azure Kinect DK experiments. Future work will include more rigorous quantitative real-world evaluation with external ground truth, broader cross-dataset evaluations, extension to other sensor configurations such as stereo or visual-inertial setups, and comparisons with a wider range of lightweight and foundation-model-based dynamic SLAM systems.

## 6. Conclusions

This paper presented SY-SLAM, a real-time RGB-D SLAM system for dynamic indoor environments that improves trajectory accuracy through a strengthened front end while preserving the mature ORB-SLAM3 back end. The proposed system combines a detector-only SuperPoint front end with ORB binary descriptors, an adaptive keypoint selection strategy, and an asynchronous YOLOv8s-based person-aware suppression module with temporal bounding-box memory. By removing keypoints inside detected person regions before ORB descriptor computation and matching, SY-SLAM reduces dynamic-feature contamination at an early stage of the pipeline while maintaining full compatibility with ORB-SLAM3’s matching, mapping, and place-recognition framework. Experiments on five dynamic TUM RGB-D fr3 sequences demonstrate that SY-SLAM significantly improves both global trajectory accuracy and local motion stability in dynamic indoor scenes. In particular, across the four walking sequences, the proposed system reduces ATE RMSE by 93.45% compared with ORB-SLAM3, while maintaining real-time performance at 46.8 Hz (21.36 ms per frame) on an Intel i9-13900H CPU with an NVIDIA RTX 4070 Laptop GPU. These results indicate that SY-SLAM achieves a practical balance among trajectory accuracy, compatibility, and runtime efficiency for dynamic indoor RGB-D SLAM in human-populated scenes.

Despite these gains, several limitations remain. The current system focuses on a single dynamic category, namely persons, and therefore may generalize less well to scenes dominated by other moving objects that are not explicitly modeled. It also relies on bounding-box-level suppression rather than refined pixel-level masks; therefore, valid static features near object boundaries may still be removed in challenging cases. In addition, the current implementation does not explicitly verify whether a detected object is actually moving, which may lead to unnecessary suppression when a detected person remains stationary for an extended period. Future work will therefore investigate multi-class dynamic suppression, lightweight motion-validation mechanisms based on optical flow or depth-geometric consistency, and more refined region filtering to preserve additional valid correspondences, especially in scenes containing non-human dynamic objects or temporarily stationary persons. It will also be valuable to evaluate the method on broader real-world and cross-dataset benchmarks and to extend the same design principles to multi-sensor configurations such as stereo-inertial and RGB-D-inertial SLAM, where cross-sensor consistency may further improve robustness and generality.

## Figures and Tables

**Table 1:** ATE results on the five TUM RGB-D fr3 sequences.

Sequence	ORB-SLAM3				SY-SLAM				Improvement Rate of RMSE (%)
	RMSE	Mean	Median	S.D.	RMSE	Mean	Median	S.D.	
fr3/w/h	0.2614	0.2164	0.2039	0.1466	0.0299	0.0248	0.0211	0.0168	**88.56**
fr3/w/r	0.7018	0.5942	0.5820	0.3734	0.0456	0.0358	0.0269	0.0283	**93.50**
fr3/w/s	0.1528	0.1422	0.1321	0.0558	0.0068	0.0060	0.0053	0.0033	**95.54**
fr3/w/x	0.4194	0.3067	0.2300	0.2861	0.0160	0.0140	0.0126	0.0077	**96.18**
fr3/s/h	0.0225	0.0183	0.0158	0.0131	0.0139	0.0122	0.0109	0.0066	**38.22**

**Table 2:** Translational RPE results on the five TUM RGB-D fr3 sequences.

Sequence	ORB-SLAM3				SY-SLAM				Improvement Rate of RMSE (%)
	RMSE	Mean	Median	S.D.	RMSE	Mean	Median	S.D.	
fr3/w/h	0.0209	0.0154	0.0111	0.0141	0.0158	0.0117	0.0095	0.0106	24.40
fr3/w/r	0.0303	0.0228	0.0168	0.0200	0.0266	0.0181	0.0137	0.0195	12.21
fr3/w/s	0.0147	0.0100	0.0066	0.0107	0.0066	0.0056	0.0048	0.0035	55.10
fr3/w/x	0.0228	0.0168	0.0122	0.0155	0.0123	0.0102	0.0086	0.0069	46.05
fr3/s/h	0.0078	0.0066	0.0057	0.0042	0.0078	0.0066	0.0058	0.0040	0.00

**Table 3:** Rotational RPE results on the five TUM RGB-D fr3 sequences.

Sequence	ORB-SLAM3				SY-SLAM				Improvement Rate of RMSE (%)
	RMSE	Mean	Median	S.D.	RMSE	Mean	Median	S.D.	
fr3/w/h	0.5337	0.4238	0.3566	0.3244	0.4106	0.3369	0.2972	0.2346	23.06
fr3/w/r	0.7055	0.5599	0.4649	0.4292	0.6017	0.4059	0.3153	0.4441	14.71
fr3/w/s	0.2988	0.2346	0.1939	0.1851	0.1757	0.1522	0.1353	0.0878	41.19
fr3/w/x	0.5371	0.3950	0.3019	0.3638	0.3967	0.2816	0.2289	0.2795	26.14
fr3/s/h	0.3646	0.3121	0.2758	0.1885	0.3588	0.3063	0.2643	0.1870	1.59

**Table 4:** Ablation study results in terms of ATE RMSE (m) on the five TUM RGB-D fr3 sequences.

Sequence	ORB-SLAM3	SuperPoint	ORB + YOLO	SuperPoint + YOLO
fr3/w/x	0.4194	0.4189	0.0171	**0.0160**
fr3/w/r	0.7018	0.5213	0.2047	**0.0456**
fr3/w/s	0.1528	0.0142	0.0074	**0.0068**
fr3/w/h	0.2614	0.3430	0.0398	**0.0299**
fr3/s/h	0.0225	0.0177	0.0260	**0.0139**

The best results are shown in bold.

**Table 5:** Runtime comparison on the TUM fr3/w/x sequence.

Method	ORB-SLAM3	SY-SLAM
Tracking thread (ms)	12.70	11.04
YOLO thread (ms)	-	2.66
Wall-clock time (ms)	20.77	21.36
Max FPS (Hz)	48.13	46.80

**Table 6:** SY-SLAM parameters used in the experiments.

Parameter	Value
RGB-D camera	Azure Kinect DK
Camera model	Pinhole
Image resolution	1280 × 720
Frame rate	30 Hz
fx,fy	609.164, 609.052
cx,cy	637.272, 367.310
Depth scale factor	1.0
Number of ORB features	1500
Number of pyramid levels	8
SuperPoint input size	1280 × 720
SP_ANMS	4
SP_ScoreThresh	0.004

**Table 7:** Literature-level comparison of SY-SLAM with representative recent dynamic-scene SLAM methods on the TUM fr3/w/x sequence.

SLAM Algorithm	ATE RMSE (m)	Dynamic-Object Perception Module	Runtime Reporting	Hardware Information
CS-SLAM [10]	0.0140	Cross-SegNet	No tracking time analysis	NVIDIA GeForce RTX 4090 16 GB RAM
SEG-SLAM [29]	0.0141	YOLOv5	No real-time performance	Intel i5-6300HQ NVIDIA GTX 960M 16 GB RAM
DynaSLAM [3]	0.0150	Mask R-CNN	No real-time performance	Intel i5 Titan X GPU
YOLOv9S [30]	0.0152	YOLOv9	Real time in some cases	NVIDIA RTX 3060 Ti
CA-SLAM [11]	0.0154	YOLOv8-SEG	No tracking time analysis	MD1 Ryzen 5 5600 6-core NVIDIA GeForce RTX 3060 GPU 32 GB RAM
**SY-SLAM** **(Ours)**	**0.0160**	**YOLOv8s**	**Real time**	**Intel Core i9 Laptop** **NVIDIA GeForce RTX 4070 Laptop** **32 GB RAM**
DFT-VSLAM [15]	0.0164	YOLOv8	No clear tracking time analysis	Intel Core i5-11500H 16 GB RAM
YOLO11-ORB-SLAM3 [31]	0.0174	YOLO11	Real time in some cases	Intel Core i5 NVIDIA GeForce RTX 5060 8 GB RAM
YOLOv5 + Clustering [32]	0.0174	YOLOv5	Real time in some cases	Intel Core i7-11700 NVIDIA RTX 3060 32 GB RAM
DS-SLAM [4]	0.0247	SegNet	No real-time performance	i7 CPU P4000 GPU 32 GB RAM
USD-SLAM [9]	0.0350	SegGPT	No real-time performance	Not reported
ORB-SLAM + YOLOv5 [33]	0.0530	YOLOv5.7-0	No tracking time analysis	NVIDIA GeForce RTX2080Ti 16 GB RAM

## Data Availability

The TUM RGB-D dataset used in this study is publicly available from the official benchmark website. The real-world experimental data supporting the findings of this study are available from the corresponding author upon reasonable request.
